# The impact and cost‐effectiveness of community‐based HIV self‐testing in sub‐Saharan Africa: a health economic and modelling analysis

**DOI:** 10.1002/jia2.25243

**Published:** 2019-03-25

**Authors:** Valentina Cambiano, Cheryl C Johnson, Karin Hatzold, Fern Terris‐Prestholt, Hendy Maheswaran, Harsha Thirumurthy, Carmen Figueroa, Frances M Cowan, Euphemia L Sibanda, Getrude Ncube, Paul Revill, Rachel C Baggaley, Elizabeth L Corbett, Andrew Phillips, for Working Group on Cost Effectiveness of HIV self‐testing in Southern Africa

**Affiliations:** ^1^ Institute for Global Health University College London London United Kingdom; ^2^ World Health Organization Geneva Switzerland; ^3^ Population Services International Washington DC USA; ^4^ Department of Global Health and Development London School of Hygiene and Tropical Medicine London United Kingdom; ^5^ Institute of Psychology, Health and Society University of Liverpool Liverpool United Kingdom; ^6^ Department of Medical Ethics and Health Policy Perelman School of Medicine University of Pennsylvania Philadelphia PA USA; ^7^ Centre for Sexual Health and HIV AIDS Research (CeSHHAR) Harare Zimbabwe; ^8^ Liverpool School of Tropical Medicine Liverpool United Kingdom; ^9^ Zimbabwe Ministry of Health and Child Care Harare Zimbabwe; ^10^ Centre for Health Economics University of York York United Kingdom; ^11^ Malawi–Liverpool–Wellcome Trust Clinical Research Programme Blantyre Malawi; ^12^ Department of Clinical Research London School of Hygiene& Tropical Medicine London United Kingdom

**Keywords:** HIV testing, community‐based HIV self‐testing, cost‐effectiveness, mathematical modelling, HIV, benefits and cost

## Abstract

**Introduction:**

The prevalence of undiagnosed HIV is declining in Africa, and various HIV testing approaches are finding lower positivity rates. In this context, the epidemiological impact and cost‐effectiveness of community‐based HIV self‐testing (CB‐HIVST) is unclear. We aimed to assess this in different sub‐populations and across scenarios characterized by different adult HIV prevalence and antiretroviral treatment programmes in sub‐Saharan Africa.

**Methods:**

The synthesis model was used to address this aim. Three sub‐populations were considered for CB‐HIVST: (i) women having transactional sex (WTS); (ii) young people (15 to 24 years); and (iii) adult men (25 to 49 years).

We assumed uptake of CB‐HIVST similar to that reported in epidemiological studies (base case), or assumed people use CB‐HIVST only if exposed to risk (condomless sex) since last HIV test. We also considered a five‐year time‐limited CB‐HIVST programme. Cost‐effectiveness was defined by an incremental cost‐effectiveness ratio (ICER; cost‐per‐disability‐adjusted life‐year (DALY) averted) below US$500 over a time horizon of 50 years. The efficiency of targeted CB‐HIVST was evaluated using the number of additional tests per infection or death averted.

**Results:**

In the base case, targeting adult men with CB‐HIVST offered the greatest impact, averting 1500 HIV infections and 520 deaths per year in the context of a simulated country with nine million adults, and impact could be enhanced by linkage to voluntary medical male circumcision (VMMC). However, the approach was only cost‐effective if the programme was limited to five years or the undiagnosed prevalence was above 3%. CB‐HIVST to WTS was the most cost‐effective. The main drivers of cost‐effectiveness were the cost of CB‐HIVST and the prevalence of undiagnosed HIV. All other CB‐HIVST scenarios had an ICER above US$500 per DALY averted.

**Conclusions:**

CB‐HIVST showed an important epidemiological impact. To maximize population health within a fixed budget, CB‐HIVST needs to be targeted on the basis of the prevalence of undiagnosed HIV, sub‐population and the overall costs of delivering this testing modality. Linkage to VMMC enhances its cost‐effectiveness.

## 1 Introduction

The ambitious UNAIDS targets, set in 2014, of diagnosing 90% of people living with HIV, having 90% of those diagnosed on antiretroviral treatment (ART) and having virological suppression in 90% of those on treatment by 2020 has prompted concerted programmatic efforts and review of progress around these three indicators 1. The annual volume of HIV tests performed in sub‐Saharan Africa (SSA) has more than doubled over ten years.

Based on UNAIDS estimates, awareness of HIV status among people living with HIV (PLHIV) continues to increase rapidly, from 45% in 2014 1 to 75% in 2017 2. Recent population‐based surveys (2015 to 2017) in Eastern and Southern African countries found that between 52% and 85% of PLHIV were aware of their status 3, 4, 5, 6, 7. These may, indeed, be underestimates, as people tend to under‐report HIV diagnosis 8. Of concern though, despite increases in HIV testing, challenges remain, as men, adolescents (10 to 19 years) and key populations remain underserved by current testing strategies 9, 10 with lower proportions diagnosed than in the general population 2. To reach the first 90 target, and possibly the even more ambitious future goals, it will be necessary to implement approaches that reach those in need of HIV testing and who are being missed.

Community‐based HIV self‐testing (CB‐HIVST), defined as the distribution of HIVST by approaches such as home distribution, mobile outreach campaigns, distribution of HIVST at workplaces, bars or educational establishments, is highly acceptable, even to populations otherwise resistant to testing 11. It provides complementary coverage to other approaches, including reaching people who have never tested before, and is reasonably accurate 12. CB‐HIVST in urban Malawi reached 68% of men aged ≥16 years and 89% of young people (16 to 29 years) within the first year of implementation 13. Similar levels of uptake were seen among men and young people through CB‐HIVST in rural Zimbabwe 14 and slightly lower in a subsequent cluster randomized controlled trial: 46.5% of men and 46.2% of people aged less than 25 15. In both Malawi and Zimbabwe, approximately a third of those who accessed CB‐HIVST reported never testing before 13, 15.

A measure of relevance for all HIV testing models is the proportion of people tested in whom the test result is positive (referred here as the test positivity rate). CB‐HIVST models report test positivity rates of approximately 8% 15 (excluding retesting while taking ART and studies that used late‐read, given the issues with the stability of the test results when reread after 72 hours), while facility‐based HIVST distribution (excluding studies that used late‐read) have found test positivity rates as high as 11% 16 and even higher rates with distribution of HIVST among female sex workers (FSW): 27% in Malawi 16 and 30% in Zimbabwe 17. However, the positivity rate may not correspond to the proportion of tests that actually result in a first diagnosis because retesting among those previously diagnosed is common in all these studies, albeit that this occurs also with standard HIV testing services (HTS) 18. The proportion of tests resulting in a first diagnosis has been shown to be an important driver of whether HIVST distribution is cost‐effective 19. As countries get closer to reaching the first “90,” the prevalence of undiagnosed HIV will decline further and test positivity rates of HIV testing models will fall, potentially impacting on whether different HIV testing approaches remain cost‐effective.

The HIV Self‐Test AfRica (STAR) project recently estimated the unit cost per individual tested across health facilities testing services 20 and CB‐HIVST sites in Zimbabwe, Malawi and Zambia 21. Using a mathematical model previously used to evaluate the potential cost‐effectiveness of HIVST 19, we aimed to identify which HIV epidemic and programmatic attributes and in which populations in SSA CB‐HIVST would have the greatest epidemiological impact, and whether CB‐HIVST could be cost‐effective, using the costs per individual tested estimated in STAR. This builds on another piece of work using the same mathematical model aimed at estimating the cost of HIV testing per diagnosis at which HIV testing programmes are cost‐effective 22.

## 2 Methods

### 2.1 Synthesis model

We used an individual‐based stochastic model of HIV transmission, progression and the effect of ART in adult populations in SSA. More detailed description of the model can be found in previous papers 19, 22, 23 and in the S1. Each time the model program is run, it samples values of variables including the number of short‐term condomless sex partners, whether they have a long‐term condomless sex partner, HIV testing, HIV acquisition, and additionally, for people with HIV, viral load, CD4 count, use of specific ART drugs, adherence to ART, resistance to specific drugs and risk of HIV‐related death, each updated in three‐month time steps from 1989.

The model provides means by which we can quantify the health effects of testing which occur via increases in the proportion of PLHIV on ART, with the consequent beneficial effects on both individual health and onward transmission. This allows estimation of the overall number of disability‐adjusted life years (DALYs) averted in the whole adult population as a result of these effects. Possible linkage to pre‐exposure prophylaxis for people tested negative is not included.

Parameters intrinsic to biological properties of HIV transmission and progression and effects of ART have been informed by data from European cohorts and confirmed by data from Africa, where available, and are kept fixed. We sampled parameter (see distributions in the Table S1) values relating to sexual behaviour, HIV transmission, HIV testing (including proportion of the population who is willing to test only if symptomatic or if HIVST is available), linkage to care and retention in care and on ART in order to generate a range of scenarios applicable to different settings in SSA (hereafter referred to as setting‐scenarios) in terms of HIV epidemic, HIV testing and ART programme characteristics.

We track a population of approximately 20,000 living adults (15 to 64 years old; increasing over time) which is then scaled up to obtain estimates relevant for a population of around nine million (in 2018, Zimbabwe 7.8 million 24, Malawi 9.8 million 25, Zambia 8.2 million 26). We excluded simulations where in 2004 there was an HIV prevalence among women below 5% or above 30% and in 2016 a number of women having condomless transactional sex (defined as having had more than three condomless sex partners in a three‐month period in the last year) below 1460 or above 146,000. These were deemed implausible given the estimates from sentinel antenatal clinics 27 and on the percentage of women who are women having transactional sex (WTS) in SSA 28. In total, 150 setting‐scenarios were obtained. Table 1 shows the characteristics of the 150 setting‐scenarios in 2017, along with examples of observed data (see S1 for more information).

**Table 1:** Characteristics of the HIV epidemic/ART programme setting‐scenarios in 2017 in SSA countries with an adult population age 15 to 64 years approximately nine million

Indicator	Median (90% range) across Setting‐scenarios (n = 150)	Examples of observed data
Population size (in million)		
Overall (15 to 64 years)	9.1 (8.2 to 9.9)	Zimbabwe 15 to 64 (2018): 7.8 million 24 Malawi 15 to 64 (2018): 9.8 million 25 Zambia 15 to 64 (2018): 8.2 million 26 Lesotho 15 to 64 (2018): 1.2 million 45
Women (15 to 49 years)	4.1 (3.8 to 4.3)	
Men (15 to 49 years)	3.9 (3.6 to 4.1)	
Young (15 to 24 years)	3.2 (3.1 to 3.2)	
Adult men (25 to 49 years)	2.3 (2.0 to 2.6)	
WTS (15 to 64 years)	0.16 (0.07 to 0.25)	
HIV prevalence		
Overall (15 to 49 years)	12.8% (4.7% to 27.5%)	Zimbabwe DHS 2015 10:14% Tanzania DHS 2011 46: 5% Uganda DHS 2011 47: 9% Lesotho DHS 2014 48: 25%
Women (15 to 49 years)	13.0% (4.5% to 29.4%)	
Men (15 to 49 years)	12.6% (5.0% to 23.3%)	
Prevalence of undiagnosed HIV		
Overall (15 to 64 years)	2.1% (0.7% to 4.8%)	Malawi PHIA 2016 4: 2.9% Zimbabwe PHIA 2016 6: 3.8% Zambia PHIA 2016 5: 4.0% Rwanda 38: approximately 0.3% (Survey estimates could be overestimates due to undisclosed diagnosed HIV 8)
Women (15 to 49 years)	1.2% (0.4% to 3.5%)	
Men (15 to 49 years)	3.3% (1.0% to 7.0%)	
HIV incidence (age 15 to 49 years) per 100 person years	0.91 (0.23 to 2.19)	Malawi PHIA 2016 4: 0.37% Zimbabwe PHIA 2016 6: 0.45% Zambia PHIA 2016 5: 0.66% Swaziland 7: 2.4% Lesotho 3: 1.5% Mbongolwane and Eshowe published in 2014 (KZN) 49: 1.2%
Number of HIV tests in year		
Overall (15 to 64 years)	2,300,000 (1,293,000 to 3,327,000)	Zimbabwe 2.2 million (2015) 50, Malawi 1.9 million (2014) 51
Women (15 to 49 years)	1,485,000 (708,000 to 2,271,000)	
Men (15 to 49 years)	796,000 (461,000 to 1,072,000)	
ANC services	721,000 (96,000 to 1,595,000)	
WTS (15 to 64 years)	78,000 (30,000 to 142,000)	
Symptomatic (PLHIV)^a^	22,000 (8,000 to 47,000)	
Symptomatic (HIV–)	181,000 (160,000 to 200,000)	
VMMC services	150,000 (114,000 to 189,000)	
Percentage of tests resulting in new HIV diagnosis^b^		
Overall (15 to 64 years)	3.2% (1.1% to 8.3%)	Observed data estimates are susceptible to bias due to rediagnosis of people who do not report previous diagnosis. 6% to 55% depending on group 52 Malawi first quarter 2016 53: 5%
Women (15 to 49 years)	2.4% (0.7% to 6.3%)	
Men (15 to 49 years)	5.1% (1.6% to 11.3%)	
Young (15 to 24 years)	2.2% (0.4% to 5.7%)	
ANC services	2.9% (0.6% to 17.5%)	
WTS (15 to 64 years)	18.0% (3.3% to 35.1%)	
Symptomatic	9.4% (3.5% to 18.2%)	
VMMC services	3.2% (0.6% to 6.9%)	
Proportion tested in past year		
Women (15 to 49 years)	29% (15% to 41%)	Zimbabwe DHS 2015 10: 49% women, 36% men (age 15 to 49 years) Namibia DHS 2013 54: 49% women, 38% men (age 15 to 49 years) Nigeria DHS 2013 55: 10% women, 10% men
Men (15 to 49 years)	19% (12% to 27%)	
Women (15 to 24 years)	25% (11% to 38%)	
Men (15 to 24 years)	17% (11% to 23%)	
When symptomatic (PLHIV)^a^^,^ c	16% (9% to 24%)	
In pregnancy (15 to 49 years)	93% (30% to 98%)	
WTS (15 to 64 years)	39% (22% to 52%)	
Proportion of HIV‐positive people diagnosed		
Overall (15 to 64 years)	83% (73% to 90%)	Malawi PHIA 2016 4: 73%; 76% in women, 67% in men Zimbabwe PHIA 2016 6: 74% Zambia PHIA 2016 5: 67% Mbongolwane and Eshowe (KZN) published in 2014 49: 75% District of Chiradzulu (rural Malawi) 2013 56: 77% Botswana 2013 to 2015 57: 78%, higher in women than men Survey estimates likely to be over‐estimates due to undisclosed diagnosed HIV 8
Women (15 to 49 years)	90% (79% to 95%)	
Men (15 to 49 years)	74% (61% to 85%)	
Women (15 to 24 years)	79% (56% to 88%)	
Men (15 to 24 years)	43% (26% to 57%)	
WTS (15 to 64 years)	75% (58% to 87%)	
Proportion of diagnosed people on ART		
Overall (15 to 64 years)	88% (59% to 92%)	Malawi PHIA 2016 4: 89% Zimbabwe PHIA 2016 6: 87% Zambia PHIA 2016 5: 85% Botswana 2013 to 15 57: 85%
Women (15 to 49 years)	89% (59% to 92%)	
Men (15 to 49 years)	87% (56% to 91%)	
Women (15 to 24 years)	89% (42% to 93%)	
Men (15 to 24 years)	79% (33% to 91%)	
WTS (15 to 64 years)	90% (50% to 94%)	
Proportion of people on ART with VL < 1000 copies/mL		
Overall (15 to 64 years)	85% (81% to 89%)	World Bank South Africa 58: 60% to 88% over districts Malawi PHIA 2016 4: 91% Zimbabwe PHIA 2016 6: 87% Zambia PHIA 2016 5: 89% District of Chiradzulu (rural Malawi) 2013 56: 91% Mbongolwane and Eshowe (KZN) published in 2014 49: 90% Rural Uganda and Kenya 59: 90% Botswana 2013 to 2015 57: 94% (among citizens of Botswana)

ANC, antenatal care; ART, antiretroviral therapy; DHS, Demographic and Health Surveys; KZN, KwaZulu‐Natal; PHIA, Population‐based HIV Impact Assessment; PLHIV, people living with HIV; VMMC, voluntary male medical circumcision; WTS, women having transactional sex.

^a^Symptoms of a WHO Stage 3 or 4 condition; ^b^This is also referred to as yield. In our model, this is the same as test positivity rate as within the Synthesis model people who received a diagnosis of HIV cannot test again, so this is the ratio between the number of new diagnoses and the number of tests performed; ^c^in this case is not in the past year but of those symptomatic/pregnant in a specific time period.

### 2.2 Implementation options under consideration

For each setting‐scenario, we projected forward for 50 years from 2018 to 2068 under seven possible CB‐HIVST implementation options (see Table 2). The reference option assumed that the current pattern and level of testing continues, including in WTS, in pregnant women (twice per pregnancy), in people presenting with potential HIV symptoms and in men presenting for voluntary medical male circumcision (VMMC), but that no CB‐HIVST is available. In the other six implementation options, HIVST is introduced through community‐based distributors in addition to the current testing in one of the following sub‐populations: young people (15 to 24 years), WTS and adult men (25 to 49 years). In these implementation options, HIVST is assumed to partially replace standard HTS (see Table 2). In our base case, CB‐HIVST implementation options involved continuous CB‐HIVST availability for the entire timeframe (50 years). We based assumptions on accuracy of CB‐HIVST on the overall results for oral fluid HIVST in a systematic review and meta‐analysis of HIV self‐test performance in field settings, including low‐ and middle‐income countries (sensitivity of 93.9% and specificity of 99.2%) 12. However, we made the conservative assumption that neither the standard HTS nor the CB‐HIVST in SSA can detect HIV within three months of infection (the time step in the model). In addition, we assumed that a positive result using a CB‐HIVST is not sufficient to make an HIV diagnosis but that a confirmatory test performed by a trained healthcare worker is required for the person to be diagnosed with HIV and be able to be linked to care and treatment. The main assumptions related to HIVST are summarized in Table 3 (and Table S2).

**Table 2:** Description of implementation options

	Core testing	Population in which CB‐HIVST is available	Possibility of using CB‐HIVST if no CLS since last test	Replacement of HTS with CB‐HIVST
Ref	Current level of testing continues, in particular testing in: General population (including WTS) In pregnant women (twice per pregnancy) In people presenting with potential HIV symptoms In men presenting for VMMC	None	Not applicable	Not applicable
1		Young people (15 to 24 years)	Yes^a^	30%^b^
2		Adult men (25 to 49 years)		30%^b^
3		WTS (15 to 64 years)		50%^d^
4		Young people (15 to 24 years)	No^c^	30%^b^
5		Adult men (25 to 49 years)		30%^b^
6		WTS (15 to 64 years)		50%^d^

CB‐HIVST, community‐based HIV self‐testing; CLS, condomless sex; HTS, HIV testing services; PLHIV, people living with HIV; VMMC, voluntary medical male circumcision; WTS, women reporting transactional sex.

^a^They can HIVST only once per year, but they can HIVST even if they had HTS in the last year (% self‐tested per year indicated in Table 4). ^b^Study offered standard HTS or HIVST and 30.9% men opted for HIVST 60. ^c^They can use HIVST only if they had condomless sex since last test (HTS or CB‐HIVST) but they can test more than once per year if having CLS. ^d^54% of women who attended a FSW clinic where provider initiated testing and counselling was available (n = 604) and were offered HIVST opted for it 17. Higher rate of substitution has been reported as well 61, 62.

**Table 3:** Assumptions on CB‐HIVST

Parameter	Value assumed for base case	Source
Sensitivity of CB‐HIVST	93.9%	12
Specificity of CB‐HIVST	99.2%	12
Sensitivity of HTS^a^	98%	63
Specificity of HTS^a^	99.2%	64
Confirmatory HTS following positive CB‐HIVST	50% by three months, 78% by one year from positive CB‐HIVST^b^	At six weeks: 50% in the arm without incentive after excluding those retesting on ART 15 Evidence on disclosure from 13 and % self‐reported linking to care in STAR
Proportion initiated on ART of those who had a positive (not previously diagnosed) CB‐HIVST	36% by three months^c^	At six weeks: 30% in the arm without incentive after excluding those retesting on ART 15
Change in condomless sex in those who are tested HIV positive by HTS	With long‐term partner: none, with short‐term partner: −17% in the first six months, −9% after	65, 66
Change in condomless sex in those tested HIV negative by HTS	No change	67 Among FSW no difference in condom use, but reduction in number of partners following HIVST at four months 68
Change in condomless sex after CB‐HIVST (and before any confirmation with HTS)	No change	Among FSW no difference in condom use, but reduction in number of partners following HIVST at four months 68

ART, antiretroviral therapy; CB‐HIVST, community‐based HIV self‐testing; HTS, HIV testing services.

^a^Assumed as facility‐based rapid diagnostic test. ^b^It is assumed that people can have a confirmatory test as a consequence of a positive CB‐HIVST only within one year of the positive CB‐HIVST. ^c^This is the median proportion initiated on ART at three months; the probability of initiating ART in people engaged to care is 0.8 per three months; for people diagnosed with HIV not linked to care by three months since diagnosis, there is a probability of linkage to care (or re‐engaging into care if lost) per three months which is sampled from a distribution 0.1 (90% range: 0.03 to 0.32).

In addition, we considered several sensitivity analyses around the implementation option of CB‐HIVST being available for adult men aged 25 to 49 years: (1) five‐year time‐limited CB‐HIVST programme; (2) assuming that the increase in the number of tests obtained by introducing CB‐HIVST is instead introduced with standard HTS (to understand, in case CB‐HIVST was not cost‐effective, whether this was due to characteristics intrinsic to CB‐HIVST or whether any increase in testing is not cost‐effective regardless of mode of testing); (3) assuming that 10% of men with negative CB‐HIVST and aged 25 to 50 link to VMMC; and (4) assuming a discount rate of 10% for both costs and health benefits. In the base case, we considered the conventional discounting rate of 3.0% per annum 29.

To reduce stochastic variability, we performed two repetitions of the projections of the population from 2018 for each implementation option in each simulation, except for the options involving WTS, where four repetitions were performed due to the small sample size of this subgroup, or in the 5‐year CB‐HIVST distribution implementation option, and we calculated the mean across these repetitions.

We assumed that all people are eligible for ART at diagnosis from 2017 and that viral load monitoring was used from mid‐2016 (at six and twelve months, and then annually).

Disability weights to calculate DALYs were derived from a comprehensive study (conditions included are: TB, WHO Stages 4 and 3) 30.

### 2.3 Costs and cost‐effectiveness approach

We used the fully loaded average recurrent cost per CB‐HIVST estimated in STAR in Zimbabwe and Malawi, respectively, US$10.18 and US$5.61 21 (see further details in the S2), and a cost per person tested for HIV testing performed by a healthcare worker (except for community‐based), derived from 31, of, respectively, US$8.66 for Zimbabwe (US$9.37 if positive) and US$4.82 for Malawi (US$5.82 if positive). Other unit costs are provided in the Table S3 but, in brief, the annual cost (including 20% of supply chain costs) of the first‐line regimen of efavirenz, lamivudine, tenofovir is US$98 per person 32, programme costs for clinic visits (not including drug or viral load or CD4 count tests) are US$20 per three months 33, 34 with an assumed reduction to US$10 per three months when viral load is measured to be <1000 copies/mL 20.

The cost‐effectiveness analysis was undertaken from the health provider perspective. Costs were estimated in 2016 US dollars. Health outcomes were quantified in DALYs averted and, as mentioned above, a discount rate of 3% was applied to both costs and health outcomes 29. We calculated incremental costs and DALYs averted for the CB‐HIVST implementation options compared with the reference over a 50‐year time horizon, in order to capture all costs and effects relevant to this decision problem. The CB‐HIVST implementation option was deemed cost‐effective if the incremental cost‐effectiveness ratio (ICER) was below US$500 per DALY averted, or if it resulted in both cost savings and DALYs averted. This use of the cost‐effectiveness threshold reflects the health foregone (opportunity costs) due to resources committed to HIV testing consequentially being unavailable to provide other interventions (i.e. so that US$500 reflects the cost‐per‐DALY‐averted of these foregone activities 35, 36). Severe constraints on overall healthcare spending in low‐income countries in the region, notably for Malawi 37 mean that this cost‐effectiveness threshold is only likely to be relevant for resource allocation within the HIV programme, which is overwhelmingly reliant on donor funds.

## 3 Results

Overall, the median (90% range) HIV prevalence across setting‐scenarios in 2017 was estimated to be 12.8% (4.7% to 27.5%), the prevalence of undiagnosed HIV (ratio between the number of PLHIV who are undiagnosed and the entire population) was 2.1% (0.7% to 4.8%) and the test positivity rate (which in our model corresponds to the proportion of tests resulting in a first diagnosis) was 3.2% (1.1% to 8.3%) (see Table 1). As expected, the test positivity was higher for women having condomless transactional sex (18.0%), adult men 15 to 49 (5.1%) and symptomatic individuals (9.4%). We modelled CB‐HIVST introduction in three independent sub‐populations: young people (aged 15 to 24 years) amounting to 3.2 million people in 2017 (35% of people aged 15 to 64 years), adult men (aged 25 to 49 years) amounting to 2.3 million men (2.0 to 2.6; 25% of people aged 15 to 64 years) and WTS (160,000 women; 70,000 to 250,000; 1.8% of people aged 15 to 64 years).

Table 4 illustrates the scope of implementation and the epidemiological impact of the considered implementation options; the highest average number of tests was required when CB‐HIVST was available continuously in the future; people self‐tested even if not exposed to risk of HIV acquisition (no sex without condom) since last test; and CB‐HIVST was available for young people (3,744,000 additional test/year compared to the reference option, +97%). Targeting adult men entailed 2,631,300 additional tests/year (+68%) and targeting WTS resulted in 222,400 additional test/year (+6%). Of note, we assumed that similar uptake of CB‐HIVST could be achieved nationally as reported for the STAR subnational demonstration projects and cluster randomized trials: respectively, 87% in young people, 73% in WTS and 71% in adult men.

**Table 4:** Mean over 50 years (2018 to 2068) of intermediate measures describing the implementation and the epidemiological impact of the options considered (across 150 setting‐scenarios)

Implementation option	Sub‐population receiving HIVST	Number of HIV tests (HTS or HIVST)/year – age 15 to 64 years (additional test compared to no intervention, relative increase)	Number of new diagnoses per year (age 15 to 49 years)	Number of new diagnoses per year in the sub‐population of interest	% tested in the past year (HTS or HIVST; age 15 to 49 years)	% tested in the past year in the sub‐population of interest	% self‐tested in the past year (age 15 to 49 years)	% self‐tested in the past year in the sub‐population of interest	% ever tested (HTS or HIVST; age 15 to 49 years)	% ever tested (HTS or HIVST) in the sub‐population of interest	% who never tested before, out of those who use HIVST for the first time	% of HIVST resulting in a diagnosis (referred to as positivity rate; age 15 to 49 years)	% of HIV‐positive people diagnosed (age 15 to 49 years)	% of HIV‐positive people diagnosed in the sub‐population of interest	% of people with HIV and VL > 1000 (out of the entire population; age 15 to 64 years)	Number of condomless (short term and long term) infectious partnership	Number of people living with HIV with VL > 1000 copies/mL	Number of deaths per year (averted compared to the no intervention)	Number of HIV infections per year (averted compared to the no intervention)	Number of additional tests per HIV infection averted (per death averted)
No Intervention	NA	3,860,300 (‐)	58,500	Young: 16,500	25%	Young: 21%	0%	Young: 0%	74%	Young: 50%	NA	NA	86%	Young: 67%	3.2%	944,500	416,900	43,300 (‐)	17,560 (‐)	‐ (‐)
				WTS: 15,400		WTS: 39%		WTS: 0%		WTS: 85%				WTS: 74%					
				Adult men: 22,900		Adult men: 21%		Adult men: 0%		Adult men: 82%				Adult men: 80%					
HIVST is available – no requirement for CLS – base case	Young people	7,604,300 (3,744,000, +97%)	55,500	18,900	51%	91%	35%	87%	98%	99%	97%	0.28%	89%	84%	2.8%	871,800	367,900	43,000 (360)	16,060 (1490)	2500 (10,460)
	WTS	4,082,800 (222,400, +6%)	55,000	16,300	26%	86%	2%	73%	79%	99%	34%	2.92%	88%	81%	2.9%	875,900	380,700	43,000 (330)	16,130 (1430)	160 (670)
	Adult men	6,481,600 (2,621,300, +68%)	57,800	24,700	42%	76%	24%	71%	81%	99.6%	21%	0.80%	91%	94%	2.7%	876,800	351,700	42,800 (520)	16,060 (1500)	1750 (5060)
HIVST is available – requirement for CLS	Young people	5,947,900 (2,087,600, +54%)	55,300	17,900	30%	35%	10%	24%	78%	54%	20%	0.61%	88%	77%	2.9%	882,700	376,800	43,000 (340)	16,160 (1400)	1490 (6070)
	WTS	4,088,400 (228,100, +6%)	54,700	15,900	26%	58%	1%	39%	78%	86%	6%	3.42%	88%	81%	2.9%	866,700	380,500	43,000 (340)	16,040 (1520)	150 (660)
	Adult men	6,150,400 (2,290,000, +59%)	57,100	23,800	33%	47%	12%	35%	78%	90%	3%	1.03%	90%	91%	2.8%	884,200	361,000	42,900 (460)	16,100 (1460)	1570 (4960)
HIVST is available, next five years	Adult men	4,082,800 (222,400, +6%)	55,700	21,800	27%	27%	3%	7%	79%	93%	25%	1.31%	88%	84%	2.9%	907,700	384,000	43,000 (310)	16,340 (1220)	180 (710)
HIVST is available – as good as HTS	Adult men	6,457,200 (2,596,900, +67%)	58,200	25,200	42%	75%	24%	70%	81%	99.6%	21%	0.92%	93%	96%	2.7%	874,400	346,900	42,800 (550)	15,970 (1590)	1640 (4760)
HIVST is available – linkage to VMMC	Adult men	6,485,800 (2,625,500, +68%)	57,100	24,200	42%	76%	24%	71%	81%	99.6%	21%	0.78%	92%	94%	2.7%	879,300	348,700	42,800 (580)	15,830 (1720)	1520 (4530)

CB‐HIVST, community‐based HIV self‐test; CLS, condomless sex; HTS, HIV testing services; NA, not applicable; VL, viral load; VMMC, voluntary medical male circumcision; WTS, women having transactional sex.

In terms of epidemiological impact, in the base case for the implementation options offering CB‐HIVST to adult men had the highest impact, with an average (across setting‐scenarios) of 1500 HIV infections averted per year, followed by targeting of young people (1490 HIV infections averted per year) and WTS (1430 HIV infections averted per year). Similarly, deaths averted (in PLHIV and without HIV) were highest when CB‐HIVST was targeted at adult men (520 death averted/year), followed by young people (360 death averted/year) and WTS (330 death averted/year). Health benefits from CB‐HIVST for adult men were enhanced if 10% of men with negative HIVST in the 25‐ to 50‐year age group link to VMMC (1720 HIV infections averted per year vs. 1500; 580 deaths averted/year vs. 520). However, in terms of numbers‐needed‐to‐test to avert one new HIV infection, targeting WTS was by far the most efficient strategy requiring 160 additional tests per HIV infection averted, compared to 2500 for young people and 1750 for adult men. For deaths, the equivalent numbers of additional tests was 670 per death averted for strategies targeting WTS, compared to 10,460 for young people and 5060 for adult men. Numbers of additional tests needed were almost halved for young people if CB‐HIVST was taken up only if they had condomless sex since their last test. Similarly, the five‐year time‐limited CB‐HIVST programme reduced the number of additional tests per HIV infection averted and per death averted to 180 and 710 respectively.

**Figure 1:**
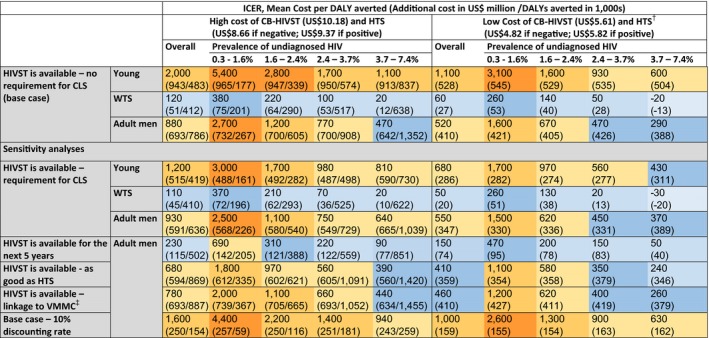
Cost per DALY averted of community‐based HIVST by implementation option, prevalence of undiagnosed HIV (quartile) and cost of testing in the sub‐population indicated – 2018 to 2068. 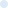
Cost‐saving; 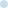
ICER $0‐$249 per DALY; 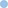
ICER $250‐$499 per DALY; 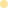
ICER $500‐$999 per DALY; 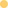
ICER $1,000‐$2,499 per DALY; 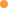
ICER ≥$2,500 per DALY; ^†^DALYs averted not reported when showing the ICERs using the cost of CB‐HIVST of $5.61 and HTS of $4.82, as the same regardless of the costs assumed; ^‡^10% of men with negative HIVST and aged 25‐50 link to circumcision; CB‐HIVST: community‐based HIVST; DALY: disability‐adjusted life years; HIVST: HIV self‐test; HTS: HIV testing services; ICER: incremental cost‐effectiveness ratio; VMMC: voluntary medical male circumcision; WTS: women having transactional sex;

Figure 1 shows the cost per DALY averted (compared to the reference option) using a 50‐year timeframe and two sets of costs for CB‐HIVST and HTS for the base case scenarios (assuming a maximum of one standards HTS annually, but regardless of sexual risk‐taking) and for several sensitivity analyses. The cost per DALY averted using a 20‐year timeframe is illustrated in Figure S1. In addition, variation in CB‐HIVST cost‐effectiveness in different settings was considered by stratifying simulations by prevalence of undiagnosed HIV (quartiles). The timeframe considered has a crucial impact on cost‐effectiveness: under the 50‐year timeframe, introduction of CB‐HIVST is cost‐effective if introduced among WTS (whether the use is limited to when having condomless sex or not), for a five‐year programme among adult men (unless the prevalence of undiagnosed HIV is below approximately 1% and cost per CB‐HIVST is US$10.18) and among adult men provided that the prevalence of undiagnosed HIV is relatively high (>3% if cost per CB‐HIVST is US$5.61, >5.5% if US$10.18). However, when considering a 20‐year time horizon, it was cost‐effective only when offered to WTS in setting with a prevalence of undiagnosed HIV above 5.5% and if the cost of CB‐HIVST was relatively low ($5.61). The cost of delivering CB‐HIVST, not surprisingly, plays a crucial role in determining the ICER. Applying higher discounting rates of 10% to additional costs and health benefits renders CB‐HIVST among adult men not cost‐effective regardless of the prevalence of undiagnosed HIV.

## 4 Discussion

CB‐HIVST offers the opportunity to reduce the “testing gap” in men, young people and WTS: subgroups that are hard to reach with standard HIV testing services. Here, we show that, when health benefits and costs are considered over a relatively long time horizon, targeted CB‐HIVST can be cost‐effective using strategies that vary by the prevalence of undiagnosed HIV. The most efficient approaches are targeted to WTS, which remain cost‐effective at all levels of prevalence of undiagnosed HIV in our setting‐scenarios (approximately 1% to 5.6%). A five‐year time‐limited CB‐HIVST programme can also be cost‐effective for adult men, at all levels when using cost for the CB‐HIVST of US$5.61 and at levels of prevalence of undiagnosed HIV above 1% when using CB‐HIVST cost of US$10.18. This is due to the fact that by considering an intervention that lasts for only five years, the cost is reduced substantially and the HIV testing earlier in time is more beneficial as the undiagnosed prevalence is declining over time. Indefinite introduction of CB‐HIVST for adult men is cost‐effective only at relatively high initial prevalence of undiagnosed HIV, depending on the cost of CB‐HIVST. When considering CB‐HIVST in WTS, it is important to note that we have assumed the same cost per person tested as in the other populations. Data on these costs have been collected as part of the STAR project but final estimates are not available yet.

Current estimates of the prevalence of undiagnosed HIV from national surveys range from 0.3% in Rwanda 38 to 4% in Zimbabwe 6 and Zambia 5 with considerable variation also within countries. For example, in Zimbabwe estimates range from 2.9% in Manicaland to 5.8% in Matabeleland South 6 and in Zambia from 1.9% in Muchinga to 5.3% in Lusaka 5. Thus, health benefits from investments at the national level can be maximized through implementation of different HIVST strategies in different geographical regions 39, 40. The corollary of this argument is that implementers may need to limit CB‐HIVST efforts, potentially through periodic campaign style implementation, in settings with very low HIV awareness, as the benefits of testing people with very low probability of being infected will be limited. Community‐based distribution of HIVST kits can take place in different ways and this partly drives the differences in costs seen in Zimbabwe compared to Malawi. The costs of government implementation CB‐HIVST may be lower than estimated in the STAR project 21 and we anticipate that delivery costs will continue to fall due to economies of scale and efficiencies from increasing familiarity with this concept. This would increase the likelihood of HIVST programmes being cost‐effective. However, the issue of “diminishing returns” will reduce the cost‐effectiveness of all HIV testing strategies as the prevalence of undiagnosed HIV falls. In the context of declining undiagnosed prevalence, programme metrics such as the cost of testing per new HIV diagnosis have potential use for monitoring programme cost‐effectiveness and in other work we have described approaches to link this metric to programme cost‐effectiveness 22.

Secondary distribution models, where HIVST kits are distributed to sexual partners of WTS or pregnant women, which have high positivity rates and similar delivery costs 41, 42, are likely to offer cost‐effective approaches to distributing HIVST 43. Additionally, improving linkage of those who test HIV negative to HIV prevention services may improve cost‐effectiveness. In our sensitivity analysis, we explored the possibility that 10% of men with a negative CB‐HIVST result are linked into VMMC and show that this would improve the benefits and cost‐effectiveness of CB‐HIVST targeted to men (considering the additional cost of VMMC). While not included in the scenarios modelled, the addition of linkage to pre‐exposure prophylaxis could also enhance impact of HIVST.

Previous cost‐effectiveness analysis of adding CB‐HIVST to existing testing services are available from urban Blantyre, Malawi 44 and for secondary distribution models, delivering self‐testing kits to sexual partners of antenatal clinic attendees in South Africa 43. The Blantyre study was in a setting of high HIV prevalence and high levels of undiagnosed and untreated PLHIV and assumed constant HIV incidence. That analysis concluded that over a 20‐year time horizon adding CB‐HIVST to facility‐based testing was cost‐effective and was suited to early ART initiation strategies. In the South African study, secondary distribution of self‐testing kits to partners of pregnant women became cost‐saving when considering the total cost of the HIV programme, although expenditure by the testing programme was increased. These findings concur with our current analysis, that community‐based strategies targeting a large group of the population, such as young people and adult men, achieve the greatest population level‐impact in terms of proportion diagnosed, but are not very cost‐effective, unless the prevalence of undiagnosed HIV is relatively high.

This work has limitations. As for any economic evaluation which takes an appropriately long‐time horizon, we rely on a mathematical model to give predictions of the long‐term impact of the alternative implementation options. We consider the implementation in three specific groups, which are either underserved by current testing approaches or characterized by a high incidence, but we could have considered slightly different groups.

## 5 Conclusions

CB‐HIVST provides a new option for reaching relatively underserved sub‐populations and can provide health benefits cost‐effectively if targeted to WTS,as well as adult men for a limited time. The prevalence of undiagnosed HIV, assumptions relating to linkage to prevention post‐HIVST and the cost of CB‐HIVST are then critical in determining whether or not wider intervention strategies, which have not only higher potential benefits but also much higher costs, should be introduced.

## Recommendations

- Targeting adult men with community‐based HIV self‐testing (CB‐HIVST) tends to allow aversion of a large number of infections as this is a large group which is currently under tested.
- Linkage to voluntary medical male circumcision (VMMC) following a negative HIVST should be considered as this can enhance the impact.
- Providing CB‐HIVST to women having transactional sex (WTS) offers the best value for money and should be implemented.
- The introduction of CB‐HIVST among adult men is cost‐effective, provided that the undiagnosed HIV prevalence is above 3% or the distribution programme is limited to 5 years duration. Shortening the intervention period improves the cost‐effectiveness because as we continue testing at the same rate the test positivity rate declines while the cost (except for discounting) remains the same.
- At its current cost, introduction of CB‐HIVST among young people does not offer value for money.
- When deciding whether to implement CB‐HIVST the overall cost of CB‐HIVST (not only the kit cost) should be considered as well as the current prevalence of undiagnosed HIV.

## Competing interest

The authors declare that they have no competing interests.

## Authors’ contributions

VC, CJ, KH, FTP, FC, PR, LC and AP substantially contributed to the formulation of research question and conceptualized the study. VC and AP worked on development and programming of the HIV synthesis model. VC performed the modelling analysis. VC and AP analysed the simulations. VC, CJ, KH, FTP, HM, TH, CF, FC, ES, GN, PR, RCB, LC and AP drafted the manuscript or critically revising it for important intellectual content. All authors gave final approval of the version to be published.

## Funding

The STAR Initiative is funded by Unitaid.

## Supporting information


**Table S1.** Parameter distributions sampled for each model run
**Table S2.** Assumptions on HIVST (extended version of Table 3)
**Table S3.** Costs included in the cost‐effectiveness evaluation
**Table S4.** Disability weights
**Figure S1.** Cost per DALY averted by implementation option, prevalence of undiagnosed HIV (quartile) and cost of testing (20 years timeframe)
**Figure S2.** HIV prevalence 15 to 49 in each setting‐scenario
**Figure S3.** HIV incidence 15 to 49 in each setting‐scenario
**Figure S4.** Mean percentage of HIVST resulting in a diagnosis (referred to as positivity rate; age 15 to 49 years)

**S1.** Synthesis model description.
