# Supplementary material for: The impact and cost‐effectiveness of community‐based HIV self‐testing in sub‐Saharan Africa: a health economic and modelling analysis
Source: J Int AIDS Soc. 2019 Mar 25;22(Suppl Suppl 1):e25243. doi: 10.1002/jia2.25243 (PMC6432108; doi:10.1002/jia2.25243)
Supplement: Supplementary file 2 — S1. Synthesis model description. [file JIA2-22-e25243-s002.docx]

S2: Supplementary Material 2

Manuscript: The impact and cost-effectiveness of community-based HIV self-testing in sub-Saharan Africa: a health economic and modelling analysis

Contents

[Parameters sampled in the Synthesis models 2](#_Toc536788387)

[Table S1. Parameter distributions sampled for each model run. 2](#_Toc536788388)

[Assumptions on HIVST 9](#_Toc536788389)

[Table S2. Assumptions on HIVST (Extended version of Table 3) 9](#_Toc536788390)

[Assumptions on costs and disability weights 10](#_Toc536788391)

[Cost of community-based HIVST 10](#_Toc536788392)

[Table S3. Costs included in the cost-effectiveness evaluation 10](#_Toc536788393)

[Table S4. Disability weights 12](#_Toc536788394)

[Additional results 13](#_Toc536788395)

[Figure S1. Cost per DALY averted by implementation option, prevalence of undiagnosed HIV (quartile) and cost of testing (20 year timeframe) 13](#_Toc536788396)

[Figure S2. HIV prevalence 15-49 in each setting scenario 14](#_Toc536788397)

[Figure S3. HIV incidence 15-49 in each setting scenario 14](#_Toc536788398)

[Figure S4. Mean percentage of HIVST resulting in a diagnosis (referred to as positivity rate; age 15-49 y) 15](#_Toc536788399)

# Parameters sampled in the Synthesis models

## Table S1. Parameter distributions sampled for each model run.

Each model run creates one setting scenario. The comparison of these setting scenarios with observed data is shown in Table 1 of the main paper.

| **Parameter name** | **Description** | **Distribution (if discrete: value; % with value)** | **Median (90% range)** | **Motivation for distribution** |
| --- | --- | --- | --- | --- |
| ***Parameters relating to sexual behaviour**** | | | | |
| *swn* | Value of multiplicative factor determining numbers of partners for those in highest new partner group (i.e. female sex workers) | Uniform(4,25) | 14.5 (5.07, 23.9) | This parameter helps to determine the extent to which the epidemic is driven by transactional sex, which is likely to vary in specific setting scenarios. |
| *eprate* | Rate of new long term partners in youngest age group (15-24). (For the other age-group the rate of new long term partners is a function of *eprate*.) | LogNormal(ln 0.1,0.25) | 0.10 (0.07, 0.15) | In order to explore epidemics with lower and greater contribution of long term partners. |
| *highsa* | Value of and fold change in multiplicative factor determining numbers of partners for those in second highest new partner group | Uniform(3,10) | 6.49 (3.34, 9.65) | Range of values that was found, in certain (randomly selected) combination with other sexual behaviour parameter values to re-produce epidemics within the observed prevalence range. Note also that sexual behaviour tends to be under-reported, particularly in women, and higher levels of behaviour have to be assumed both to be consistent with levels of risk behaviour reported in men, and to generate an epidemic of the proportions observed (e.g.[1, 2]). |
| *p_rred_p* | Proportion of the population in whom the sexual risk behaviour is very low | Uniform(0.1,0.5) | 0.30 (0.12, 0.48) | In order to include a person-level effect on sexual behaviour this and the parameter below allow the population to be divided into three according to the lifelong tendency to have condomless sex. |
| *p_hsb_p* | Proportion of the population in whom the sexual risk behaviour has a tendency to be higher than average | Uniform(0, 0.495) | 0.247 (0.025, 0.470) | As above |
| *newp_factor* | Overall average level of sexual risk behaviour. The correlation with the above parameters induced by the sampling of this parameter is to provide a focus on parameter space most likely to give low values of the overall fit. For example, if the sampling of *swn* and *highsa* give values at the high end of the distribution and sampling of *p_rred_p* produces a value at the low end then the model simulation run will produce an epidemic which is too large, unless there is some compensation when selecting the value of this parameter. | 3 x (6.5/highsa)x(14.5/swn) x (p_rred_p/0.3) x (0.025/p_hsb_p) x exp(Normal(0, 0.5^2^) | 5.03 (0.87, 64.3) | See description of parameter |
| *conc_ep* | Parameter indicating the degree to which those with a long term condomless sex partner have a lower of higher probability of short term condomless sex partners than those without a long term condomless sex partner. | LogNormal(0,0.6) | 1 (0.37, 2.66) | This is likely to vary across setting scenarios and we wished to consider across the range. Again, this distribution of values was found, in certain (randomly selected) combination with other sexual behaviour parameter values to re-produce epidemics within the observed prevalence range. |
| *ych_risk_beh_newp* | Degree of reduction in condomless sex with short term partners per year for 1995 – 1998. From 1998 to 2005 this degree of reduction is divided by 3 | Uniform(0,0.20) | 0.10 (0.01, 0.19) | In order to explain the decrease in incidence and prevalence of HIV in southern Africa in the late 1990s it is necessary to assume there was a reduction in condomless sex, which is supported by data in Zimbabwe, for example [3, 4] |
| *ych_risk_beh_ep* | Degree of reduction in condomless sex per year with long term partners from 1995 - 2000 | Uniform(0,0.20) | 0.10 (0.01, 0.19) | As above |
| *ch_risk_diag_newp* | Degree of reduction (fold change) in condomless sex with short term partners in a person diagnosed with HIV for the first 6 months since diagnosis. Afterwards the square root of *ch_risk_diag_newp* applies | Beta(12,2) | 0.87 (0.69, 0.97) | Informed by [5] |
| *ych2_risk_beh_newp* | Degree of change in condomless sex with short term partners per year from 2010 – 2015 | Uniform(-0.04,0.04) | 0 (-0.036, 0.036) | It is uncertain whether there have been recent changes in condomless sex, hence a neutral distribution was used. |
| *p_ccufsw_base* | Base rate per 3 months of becoming a sex worker who consistently use condom (for women 15-30). This applies from 1995 when reduction in sexual behaviour starts due mainly to availability of condoms. This is multiplied by 0.33 in women aged 30 to 40 and 0.2 for those over 40 years old. | Uniform(0,0.002) | 0.001 (0.0001, 0.0019) | There is a lot of uncertainty on the number of FSW who consistently use condom and this varies by setting. |
| **Parameters relating to transmission*** | | | | |
| *fold_tr* | Multiplicative factor determining the uncertainty in transmission rate in different viral load groups | LogNormal(ln 1, 0.3) | 1 (0.61, 1.64) | To reflect uncertainty in the transmission rate for a given viral load. |
| *tr_rate_undetec_vl* | HIV transmission rate for individuals with VL < 2.7 log cps/mL | LogNormal(ln 0.001, 1.5)  [Truncated at 1] | 0.001 (0.00008, 0.003) | To reflect the uncertainty in the transmission rate when viral load is undetectable |
| *fold_change_w* | The fold difference in female to males transmission rate compared with male to female, for a given viral load. | LogNormal(ln 1.5, 0.3) | 1.5 (0.91, 2.44) | Informed by the higher incidence and prevalence in women and some direct evidence. |
| *fold_change_sti* | Multiplicative factor accounting for higher rate of acquisition in people with STI | LogNormal(ln 3, 0.3) | 3.00 (1.84, 4.91) | To reflect uncertainty in magnitude of this effect. |
| *res_trans_factor* | Parameter determining the probability that if NNRTI resistance mutation present in source partner that this is not present/detectable in virus new host (In this version they are multiplied by 0.2) | 1(so 80% chance of transmission): 33%  0.75 (so 85% chance of transmission): 33%  0.5 (so 90% chance of transmission): 33% | 0.75 (0.5, 1.0) | Informed by the values needed to lead to the range of transmitted NNRTI resistance observed [6-8] |
| **Parameters relating to HIV testing*** | | | | |
| *p_hard_reach_w* | Proportion of the female population considered hard to reach and that cannot be tested for HIV unless they develop symptoms or if HIVST is available | Uniform(0.05,0.3) | 0.175 (0.062, 0.287) | Wide range required in order to have a relatively wide distribution for the proportion of people unaware of their HIV status in 2017 |
| *hard_reach_higher_in_men* | Thi parameter is added to *p_hard_reach_w* to determine the proportion of men hard to reach | Uniform(0,0.15) | 0.075 (0.007, 0.142) | The proportion ever tested is consistently higher in women than men. |
| *base_test_incr* | Parameter determining the rate of quadratic increase in the baseline rate of HIV testing (it applies until 2012) | LogNormal (0, 0.6) | 1.00 (0.37, 2.67) | Wide range required in order to have a relatively wide distribution for the proportion of people unaware of their HIV status in 2017 |
| *an_lin_incr_test* | Annual increase in age and gender specific HIV testing rate per 3 month during the linear increase (any testing outside ANC; it applies between 2012 and 2018) | Uniform(0,0.015) | 0.007 (0.0007, 0.0142) | Range and pattern required to re-produce the observed range in proportion of HIV positive people diagnosed (see Table 4 of main paper). |
| *rate_testanc_inc* | Parameter determining the rate of quadratic increase in testing in ANC clinics between 1994 and the end of 2016 | Beta'(2.5,900) | 0.0024 (0.0006, 0.0061) | Government of Malawi Ministry of Health Quarterly Reports. Again distribution is intended to reflect variation across setting scenarios. |
| *incr_test_rate_sympt* | Relative increase each 3 months in the probability of a person with a WHO stage 3 or 4 disease to be tested for HIV between 1997 and the end of 2014. It is truncated so that the maximum is 0.8. | Uniform(1.03,1.07) | 1.05 (1.032, 1.068) | Little direct data on this parameter and wide range taken to reflect uncertainty and variation across settings. |
| **Parameters relating to pre-ART care and progression of HIV*** | | | | |
| *fx* | Multiplicative factor to alter the average rate of CD4 count decline in natural HIV progression (which thus alters the incubation period distribution). | LogNormal(1.0,0.20) | 1.00 (0.72, 1.39) | Derived based on consideration of evidence from natural history studies [9-17] |
| *fold_change_ac_death_rate* | Fold change in all cause death rate compared to base rate | 1: 33%  1.5: 33%  2: 33% | 1.5 (1.0, 2.0) | To reflect variation in non-AIDS death rate by setting (base case is for South Africa) |
| *prob_loss_at_diag* | Probability that a person (without current WHO stage 3 or 4 disease) is immediately lost after initial HIV diagnosis (so not linked to care by 3 month) . | Beta(2.5,10) | 0.2 (0.05, 0.4) | [18] |
| *prob_lossdiag_adctb* | Probability that a person with AIDS or acute TB is immediately lost after initial HIV diagnosis (so not linked to care by 3 month) | Beta(5,95) | 0.05 (0.02, 0.09) | To reflect uncertainty and variability across settings |
| *prob_lossdiag_who3e* | Probability that a person with current WHO stage 3 condition is immediately lost after initial HIV diagnosis (so not linked to care by 3 month) | Beta(15,85) | 0.15 (0.10, 0.21) | To reflect uncertainty and variability across settings |
| *rate_lost* | For people under care yet to start ART or previously have taken ART, the rate of being lost to care per 3 months. | LogNormal (ln 0.02, 0.4) | 0.02 (0.01, 0.04) | Uncertain and will vary by setting. Distribution chosen to reflect this. This is one of the parameters influencing the proportion of diagnosed people on ART. |
| *rate_return* | Probability of return to care for a person who has been diagnosed with HIV (and may have started ART) but is now lost and not on ART, without current WHO stage 3 or 4 disease, per 3 months. | LogNormal (ln 0.1, 0.7) | 0.1 (0.03, 0.32) | As above |
| *prob_return_adc* | Probability of return to care for a person who has been diagnosed with HIV (and may have started ART) but is now lost and not on ART and has a WHO stage 4 condition. This is a probability that operates just for the 3 month period that the events occurs. | Beta(12,8) | 0.60 (0.42, 0.77) | As above |
| *rate_loss_persistence* | Rate of loss from majority virus of transmitted resistance mutations (per 3 months) | Uniform(0.005,0.02) | 0.013 (0.006, 0.02) | [18-21] |
| **Parameters relating to people on ART** | | |  |  |
| *an_incr_pr_art_init* | Factor multiplied by the square of the years since ART introduction to obtain the probability of ART initiation per 3 months (it applies to people diagnosed with HIV ART-naïve). It is truncated at 0.8 | Beta(1.2,100) | 0.01 (0.0009, 0.03) | These parameters contribute to determine the proportion of HIV diagnosed people who are on ART. The distributions are chosen such that combinations of these parameters lead to observed proportions of HIV diagnosed people on ART (e.g. Population Health Impact Surveys[22-26]) |
| *prob_lost_art* | For a person who interrupts / stops ART the probability that they are simultaneously lost from care. | Beta(4,4) | 0.50 (0.23, 0.77) | [27-29] |
| *rate_restart* | Rate of restart of ART for people who previously have been on ART and have returned to care, per 3 months. | LogNormal (ln 0.5, 0.5) | 0.50 (0.22, 1.14) | [29] Assumed to be high, given the person has returned to care. Most people who are regularly seen in clinics who have previously started ART are on ART. |
| *adh_pattern* | Population adherence profile; described in terms of the proportion having a given average adherence and period-to-period variability in adherence. | 1 33.3%  2 33.3%  99 33.3% | NA | Reflection of wide range of adherence profiles in different settings, informed by differences in proportions of people on ART with viral load suppression. |
| *rate_int_choice* | Rate of interruption / stopping of ART per 3 months. Also influenced by current drug toxicity and underlying tendency to adhere. | LogNormal(ln 0.003,0.5) | 0.003 (0.001, 0.007 | [27-29] |
| *red_adh_tb_adc* | Factor subtracted from the adherence level in 3 month periods with acute TB or when AIDS develops | LogNormal (ln 0.1, 0.5) | 0.10 (0.04, 0.23) | To reflect uncertainty in magnitude of this effect |
| *red_adh_tox_pop* | Amount by which toxicity is assumed to affect adherence (it is assumed that this is the case in 30% of periods in which toxicity is present | LogNormal (ln 0.05, 0.5) | 0.05 (0.02, 0.11) | To reflect uncertainty in magnitude of this effect |
| *add_eff_adh_nnrti* | Factor added to the adherence level in people on NNRTI (due to the longer half life of NNRTI) | LogNormal (ln 0.10, 0.30) | 0.10 (0.06, 0.16) | To reflect uncertainty in magnitude of this effect |
| *altered_adh_sec_line_pop* | Altered adherence for those on second line | LogNormal(ln 0.05, 0.05) | 0.05 (-0.03, 0.13) | To reflect uncertainty in magnitude of this effect |
| *incr_rate_int_low_adh* | Parameter indicating the extent to which people with a long term average adherence in the lowest group have a multiplicatively increased risk of ART interruption. | Uniform(1,2) | 1.50 (1.05, 1.95) | Low adherence predicts interruption of ART (unpublished data) |
| *pr_switch_line* | Probability of switch to second line per 3 months in a person who has fulfilled the failure criteria for first line failure. | Beta(2,100) | 0.02 (0.004, 0.05) | [30, 31] In several settings, including Zimbabwe, the proportion of people who have started second line ART is consistent with a value for *pr_switch*_line of below 0.1 (e.g. Lesotho, Malawi) (Government of Malawi MoH Quarterly Reports). |
| *clinic_not_aw_int_frac* | If a person interrupts ART, the probability that this is not disclosed to the clinic and they are classified as being on ART | Beta(6,4) | 0.61 (0.34, 0.83) | Uncertain and will vary by setting, hence a broad distribution. |

Model runs are not accepted as “setting scenarios” if HIV prevalence among women in in 2004 is below 5% or above 30% or if in 2016 the number of women having condomless transactional sex (defined as having had more than 3 condomless sex partners in a 3 month period in the last year) below 1,460 or above 146,000.

* Further details of modelling of demographics, sexual behaviour, HIV transmission and HIV testing and associated parameters are explained in detail in a separate supplement.

# Assumptions on HIVST

## Table S2. Assumptions on HIVST (Extended version of Table 3)

| **Parameter** | **Value assumed for base case** | **Source** |
| --- | --- | --- |
| Sensitivity of CB-HIVST | 93.9% | [32] |
| Specificity of CB-HIVST | 99.2% | [32] |
| Sensitivity of HTS* | 98% | [33] |
| Specificity of HTS* | 99.2% | [34] |
| Failure at performing CB-HIVST^ | 0.3% | [32] |
| Failure at performing HTS*** | 0.15% | [35] |
| Confirmatory HTS following positive CB-HIVST | 50% by 3 months,  78% by 1 year from positive HIVST.** | At 6 week: 50% in the arm without incentive after excluding those re-testing on ART [36]  Evidence on disclosure from [37, 38] and % self-reported linking to care in STAR |
| Proportion initiated on ART of those who had a positive CB-HIVST (not previously diagnosed) | 36% by 3 months | At 6 week: 30% in the arm without incentive after excluding those re-testing on ART [36] |
| Change in condomless sex in those who are tested HIV+ by HTS | with long-term partner: -none,  with short-term partner: -17% in the first 6 months, -9% after | [5, 39] |
| Change in condomless sex in those tested HIV- by HTS | No change | [40]  Among FSW no difference in condom use, but reduction in number of partners following HIVST at 4 months [41] |
| Change in condomless sex after HIVST (and before any confirmation with HTS) | No change | Among FSW no difference in condom use, but reduction in number of partners following HIVST at 4 months [41] |
| Negative psychological consequences in people receiving positive HIVST | QoL of 0.8 in 3% of people receiving positive HIVST | 0.1% based on 200,000 kits [42] |

ART: antiretroviral therapy; CB-HIVST: community-based HIV self-testing; HTS: HIV testing services; QoL: Quality of Life;

*assumed as facility based rapid diagnostic test; ** It is assumed that people can have a confirmatory test as a consequence of a positive HIVST only within 1 year of the positive HIVST. ^If people fail at performing the HIVST, the cost of HIVST is incurred but the people do not obtain a result; *** if health worker fail at performing HTS, the cost of an additional test is incurred but the test is repeated so the person will know the result of the test;

# Assumptions on costs and disability weights

## Cost of community-based HIVST

The costs included in the analysis for CB-HIVST and standard HTS are fully loaded recurrent costs, including all demand-generation, supply chain, fuel, wastage, human resources in administering the test as well as the kit itself, but without overheads and start-up costs. Although our settings scenarios reflect diversity in epidemics and programmes across SSA , we derive our costings based on studies conducted in three specific countries: Zimbabwe, Malawi and Zambia [26]. Assuming a kit cost of US$2 [36], the average recurrent cost per CB-HIVST was US$10.18 in Zimbabwe, US$5.61 in Malawi and US$15.30 in Zambia [26]. The difference in these costs partly reflects the difference in staff cost in these countries and how CB-HIVST were delivered. In Zimbabwe, CB-HIVST was delivered by community-based distribution agents (CBDAs) over 4-6 week campaigns per each of 8 districts and was very labour intensive. In Malawi fewer CBDAs (one seventh) were used to deliver HIVST throughout the year [26]. For the forms of HIV testing performed by a health care worker (except for community-based) the cost per person tested negative and positive estimated in the facilities (derived from [37]) were: US$8.66 in Zimbabwe (US$9.37 if positive), US$4.82 in Malawi (US$5.82 if positive) and US$4.12 in Zambia (US$5.72 if positive) .

## Table S3. Costs included in the cost-effectiveness evaluation

| **Item** | **Unit Cost (US$)** | **Source / explanation** |
| --- | --- | --- |
| Community-based HIVST (per person tested) - Zimbabwe | US$10.18 | Paper on costing in same issue |
| Community-based HIVST (per person tested) - Malawi | US$5.61 | Paper on costing in same issue |
| Facility-based negative HTS ( applied to HIV testing pre-VMMC, HIV testing performed in ANC,…) if the result is negative - Zimbabwe | US$8.66 | Derived from [43] |
| Facility-based negative HTS (applied to HIV testing pre-VMMC, HIV testing performed in ANC,..) if the result is negative - Malawi | US$4.82 | Derived from [43] |
| Facility-based negative HTS ( applied to HIV testing pre-VMMC, HIV testing performed in ANC,…) if the result is positive - Zimbabwe | US$9.37 | Derived from [43] |
| Facility-based negative HTS (applied to HIV testing pre-VMMC, HIV testing performed in ANC,..) if the result is positive - Malawi | US$5.82 | Derived from [43] |
| Community-based negative HTS | US$24.5 | [44] |
| Additional cost for HTS test if the result is positive (applied to community based-testing | US$0.71 | Derived from[38] |
| Drug per year (supply chain):  First-line: tenofovir/3TC/efavirenz  Second-line: zidovudine/3TC/atazanavirl | US$82 (+20%)  US$245 (+20%) | [45] |
| Treatment of a WHO stage 4 condition over 3 months (cost is incurred for 3 months)  Treatment of a WHO stage 3 condition over 3 months (cost is incurred for 3 months)  Treatment of TB per 3 months (cost is incurred for 6 months)  Cotrimoxazole annual cost | US$200  US$20  US$50  US$5 | Specific data not available on average unit costs of treating WHO stage 3 and 4 conditions and per clinic visit costs - costs used are informed by evidence synthesis from studies that cost according to current CD4 count of those in pre-ART care, cost of ART initiation, which also include costs of CD4 tests [46] |
| CD4 count measurement | US$10 | [47, 48] |
| Viral load measurement: | US$22 | Human resource costs $3, sample collection consumables $2, relaying of results $2 (this costing information was provided by Medecin Sans Frontiers (MSF) (K Bonner), with the most recent information update February 2014), running the test (including equipment and other costs such as consumables, maintenance and shipping) $15 (http://www.theglobalfund.org/en/mediacenter/newsreleases/2015-06-10_New_Approach_on_HIV_Viral_Load_Testing/ http://www.theglobalfund.org/en/procurement/viral-load-early-infant-diagnostics/) |
| Non-ART programme per year, $40 per year if on tiered care due to viral load < 1000 | US$80 | [49, 50]  Bill and Melinda Gates Foundation tiered care meeting report (the per client cost of running the Khayelitsha adherence clubs was $58 per client per year compared to standard clinic care of $108 per client per year. At the Infectious Disease Institute in Kampala, the annual costs per client for physician, nurse, and pharmacy only visits were $60, $45, and $19, respectively. |
| Targeted adherence counselling intervention triggered by a viral load > 1000 copies/mL | US$20 | Author assumption |
| VMMC | US$61 | [51] |

* It is conceived of as the fully loaded costs of a test being done, including any demand generation, supply chain, fuel, wastage, human resources in administering the test as well as the kit itself.VMMC: voluntary medical male circumcision;

## Table S4. Disability weights

Values are 1 in each three month period except for the following:

| **Condition in current 3 month period** | **Disability weight for current 3 month period** | **Source** |
| --- | --- | --- |
| Any WHO stage 3 condition (except TB) in current 3-month period | 0.78 | [52] |
| TB in current 3-month period | 0.60 | [52] |
| Any WHO stage 4 condition in current 3-month period | 0.46 | [52] |

# Additional results

## Figure S1. Cost per DALY averted by implementation option, prevalence of undiagnosed HIV (quartile) and cost of testing (20 year timeframe)

| **ICER, Mean Cost per DALY averted (Additional cost in million /DALYs averted in 1,000s)** | | **High cost of CB-HIVST ($10.18) and HTS**  **$8.66 if negative; $9.37 if positive)** | | | | | **Low cost of CB-HIVST ($5.61) and HTS***  **($4.82 if negative; $5.82 if positive)** | | | | | |  |
| --- | --- | --- | --- | --- | --- | --- | --- | --- | --- | --- | --- | --- | --- |
|  |  | **Overall** | **Prevalence of undiagnosed HIV** | | | | **Overall** | **Prevalence of undiagnosed HIV** | | | | |  |
|  |  |  | **0.3 - 1.6%** | **1.6 – 2.4%** | **2.4 – 3.7%** | **3.7 – 7.4%** |  | **0.3 - 1.6%** | **1.6 – 2.4%** | **2.4 – 3.7%** | **3.7 – 7.4%** |  |  |
| **HIVST is available – no requirement for CLS (base case)** | **Young** | 5,400  (536/100) | 12,600  (531/42) | 6,300  (532/85) | 4,400  (541/123) | 3,600  (541/148) | 3,200  (318) | 7,300  (310) | 3,700  (312) | 2,600  (323) | 2,200  (326) | |  |
|  | **WTS** | 900  (65/72) | 1,900  (64/34) | 1,200  (65/52) | 660  (68/103) | 620  (63/101) | 720  (52) | 1,500  (52) | 1,000  (52) | 540  (55) | 490  (50) | |  |
|  | **Adult men** | 1,800  (381/210) | 4,600  (390/84) | 2,400  (381/158) | 1,500  (381/247) | 1,100  (372/346) | 1,200  (243) | 2,800  (235) | 1,500  (236) | 1,000  (249) | 720  (250) | |  |
| **Sensitivity analyses** | | | | | | | | | | | | | |
| **HIVST is available – requirement for CLS** | **Young** | 4,000  (302/75) | 8,000  (276/34) | 8,500  (285/34) | 2,500  (288/115) | 3,100  (358/116) | 2,500  (185) | 4,900  (170) | 5,100  (173) | 1,600  (181) | 1,900  (216) | |  |
|  | **WTS** | 970  (63/65) | 2,100  (63/30) | 1,200  (65/56) | 670  (60/90) | 760  (65/86) | 770  (51) | 1,700  (51) | 950  (53) | 530  (48) | 590  (50) | |  |
|  | **Adult men** | 1,200  (324/148) | 5,400  (306/57) | 2,300  (316/137) | 1,700  (303/176) | 1,700  (369/220) | 1,400  (206) | 3,300  (189) | 1,400  (197) | 1,100  (198) | 1,100  (238) | |  |
| **HIVST is available for the next 5 years** | **Adult men** | 800  (144/181) | 2,000  (143/72) | 1,000  (142/138) | 710  (151/213) | 480  (142/298) | 580  (105) | 1,400  (99) | 730  (101) | 530  (113) | 360  (107) | |  |
| **HIVST is available - as good as HTS** |  | 1,300  (335/256) | 4,000  (334/84) | 1,800  (334/185) | 1,000  (342/337) | 790  (330/417) | 860  (220) | 2,400  (206) | 1,200  (214) | 690  (232) | 550  (230) | |  |
| **HIVST is available – linkage to VMMC~** |  | 1,600  (391/246) | 4,000  (408/103) | 2,100  (393/187) | 1,300  (391/301) | 950  (372/391) | 1,000  (253) | 2,500  (253) | 1,300  (249) | 860  (258) | 640  (251) | |  |
| **Base case – 10% discounting rate** |  | 2,400  (212/90) | 5,700  (215/38) | 3,200  (211/66) | 2,000  (211/108) | 1,400  (210/145) | 1,500  (138) | 3,500  (132) | 2,000  (133) | 1,300  (140) | 1,000  (145) | |  |

•Cost-saving; •ICER $0-$249 per DALY; •ICER $250-$499 per DALY; •ICER $500-$999 per DALY; •ICER $1,000-$2,499 per DALY; •ICER ≥$2,500 per DALY;

~10% of men with negative HIVST and aged 25-50 link to circumcision; *DALYs averted not reported when showing the ICERs using the cost of CB-HIVST and HTS as in Malawi as the same regardless of the costs assumed;HTS: HIV testing services; VMMC: voluntary medical male circumcision;

## Figure S2. HIV prevalence 15-49 in each setting scenario


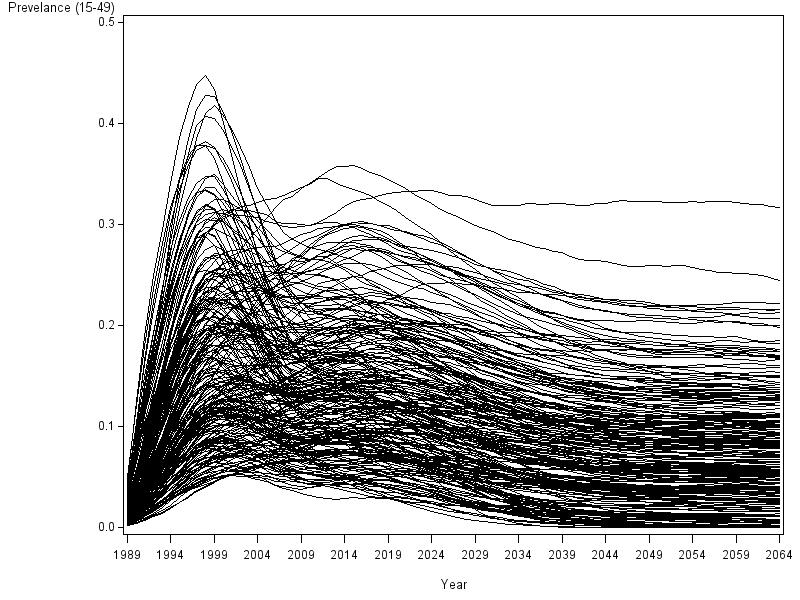


## Figure S3. HIV incidence 15-49 in each setting scenario


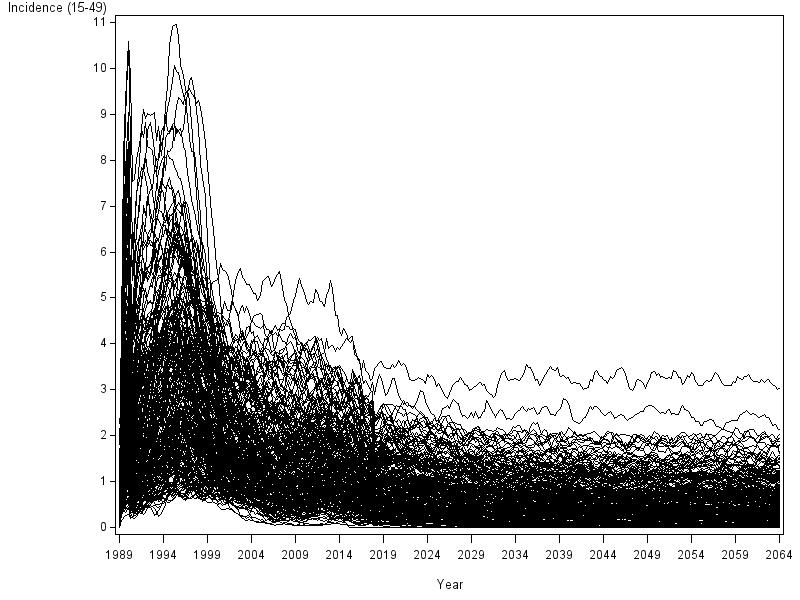


## Figure S4. Mean percentage of HIVST resulting in a diagnosis (referred to as positivity rate; age 15-49 y)

We considered the three base implementation optios: in young, WTS and adult men


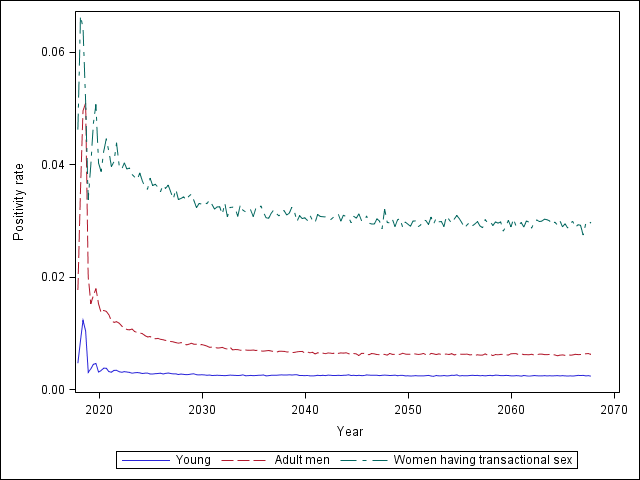


1. Johnson LF, Dorrington RE, Bradshaw D, Pillay-Van Wyk V, Rehle TM. Sexual behaviour patterns in South Africa and their association with the spread of HIV: Insights from a mathematical model. Demogr Res. 2009;21:289-339.

2. Gregson S, Zhuwau T, Ndlovu J, Nyamukapa CA. Methods to reduce social desirability bias in sex surveys in low-development settings - Experience in Zimbabwe. Sex Transm Dis. 2002;29(10):568-75.

3. Gregson S, Gonese E, Hallett TB, Taruberekera N, Hargrove JW, Lopman B, et al. HIV decline in Zimbabwe due to reductions in risky sex? Evidence from a comprehensive epidemiological review. Int J Epidemiol. 2010;39(5):1311-23.

4. Halperin DT, Mugurungi O, Hallett TB, Muchini B, Campbell B, Magure T, et al. A Surprising Prevention Success: Why Did the HIV Epidemic Decline in Zimbabwe? Plos Med. 2011;8(2).

5. Fonner VA, Denison J, Kennedy CE, O'Reilly K, Sweat M. Voluntary counseling and testing (VCT) for changing HIV-related risk behavior in developing countries. Cochrane Database Syst Rev. 2012;9:CD001224.

6. Afonso JM, Bello G, Guimaraes ML, Sojka M, Morgado MG. HIV-1 genetic diversity and transmitted drug resistance mutations among patients from the North, Central and South regions of Angola. PLoS One. 2012;7(8):e42996.

7. Rowley CF, MacLeod IJ, Maruapula D, Lekoko B, Gaseitsiwe S, Mine M, et al. Sharp increase in rates of HIV transmitted drug resistance at antenatal clinics in Botswana demonstrates the need for routine surveillance. J Antimicrob Chemother. 2016;71(5):1361-6.

8. National Institute for Communicable Diseases DotNHLS. Communicable Disease Communiqué. Prospective sentinel surveillance of human immunodeficiency virus related drug resistance. . 2016. Contract No.: 3.

9. Pantazis N, Touloumi G, Walker AS, Babiker AG. Bivariate modelling of longitudinal measurements of two human immunodeficiency type 1 disease progression markers in the presence of informative drop-outs. Journal of the Royal Statistical Society: Series C (Applied Statistics). 2005;54(2):405-23.

10. Sabin CA, Devereux H, Phillips AN, Hill A, Janossy G, Lee CA, et al. Course of viral load throughout HIV-1 infection. J Acquir Immune Defic Syndr. 2000;23(2):172-7.

11. JB H, M B, E D, C T, C D, J LC, et al. - Natural history of serum HIV-1 RNA levels in 330 patients with a known date of. D - 8710219. (- 0269-9370 (Print)):- 123-31.

12. O'Brien TR, Rosenberg PS, Yellin F, Goedert JJ. Longitudinal HIV-1 RNA levels in a cohort of homosexual men. J Acquir Immune Defic Syndr Hum Retrovirol. 1998;18(2):155-61.

13. Henrard DR, Phillips JF, Muenz LR, Blattner WA, Wiesner D, Eyster ME, et al. Natural history of HIV-1 cell-free viremia. JAMA. 1995;274(7):554-8.

14. Lyles RH, Munoz A, Yamashita TE, Bazmi H, Detels R, Rinaldo CR, et al. Natural history of human immunodeficiency virus type 1 viremia after seroconversion and proximal to AIDS in a large cohort of homosexual men. Multicenter AIDS Cohort Study. J Infect Dis. 2000;181(3):872-80.

15. Touloumi G, Pantazis N, Babiker AG, Walker SA, Katsarou O, Karafoulidou A, et al. Differences in HIV RNA levels before the initiation of antiretroviral therapy among 1864 individuals with known HIV-1 seroconversion dates. AIDS. 2004;18(12):1697-705.

16. Koot M, Keet IP, Vos AH, de Goede RE, Roos MT, Coutinho RA, et al. Prognostic value of HIV-1 syncytium-inducing phenotype for rate of CD4+ cell depletion and progression to AIDS. Ann Intern Med. 1993;118(9):681-8.

17. Mellors JW, Munoz A, Giorgi JV, Margolick JB, Tassoni CJ, Gupta P, et al. Plasma viral load and CD4+ lymphocytes as prognostic markers of HIV-1 infection. Ann Intern Med. 1997;126(12):946-54.

18. Rosen S, Fox MP. Retention in HIV care between testing and treatment in sub-Saharan Africa: a systematic review. Plos Med. 2011;8(7):e1001056.

19. Castro H, Pillay D, Cane P, Asboe D, Cambiano V, Phillips A, et al. Persistence of HIV-1 transmitted drug resistance mutations. J Infect Dis. 2013;208(9):1459-63.

20. Jain V, Sucupira MC, Bacchetti P, Hartogensis W, Diaz RS, Kallas EG, et al. Differential persistence of transmitted HIV-1 drug resistance mutation classes. J Infect Dis. 2011;203(8):1174-81.

21. Yang WL, Kouyos RD, Boni J, Yerly S, Klimkait T, Aubert V, et al. Persistence of transmitted HIV-1 drug resistance mutations associated with fitness costs and viral genetic backgrounds. PLoS Pathog. 2015;11(3):e1004722.

22. ICAP at Columbia University. Lesotho population-based HIV impact assessment LePhia 2016–2017. 2017.

23. ICAP at Columbia University. Malawi population-based HIV impact assessment MPHIA 2015–2016. 2016.

24. ICAP at Columbia University. PHIA Project [Available from: <http://phia.icap.columbia.edu/resources/>.

25. ICAP at Columbia University. Zambia population-based HIV impact assessment ZAMPHIA 2015–2016. 2016.

26. ICAP at Columbia University. Zimbabwe population-based HIV impact assessment ZIMPHIA 2015–2016. 2016.

27. McMahon JH, Spelman T, Ford N, Greig J, Mesic A, Ssonko C, et al. Risk factors for unstructured treatment interruptions and association with survival in low to middle income countries. AIDS Res Ther. 2016;13:25.

28. Kranzer K, Lewis JJ, Ford N, Zeinecker J, Orrell C, Lawn SD, et al. Treatment interruption in a primary care antiretroviral therapy program in South Africa: cohort analysis of trends and risk factors. J Acquir Immune Defic Syndr. 2010;55(3):e17-23.

29. Kranzer K, Ford N. Unstructured treatment interruption of antiretroviral therapy in clinical practice: a systematic review. Trop Med Int Health. 2011;16(10):1297-313.

30. Fox MP, Cutsem GV, Giddy J, Maskew M, Keiser O, Prozesky H, et al. Rates and predictors of failure of first-line antiretroviral therapy and switch to second-line ART in South Africa. J Acquir Immune Defic Syndr. 2012;60(4):428-37.

31. Johnston V, Fielding KL, Charalambous S, Churchyard G, Phillips A, Grant AD. Outcomes following virological failure and predictors of switching to second-line antiretroviral therapy in a South African treatment program. J Acquir Immune Defic Syndr. 2012;61(3):370-80.

32. Figueroa C, Johnson C, Ford N, Sands A, Dalal S, Meurant R, et al. Reliability of HIV rapid diagnostic tests for self-testing compared with testing by health-care workers: a systematic review and meta-analysis. Lancet HIV. 2018.

33. Pant Pai N, Balram B, Shivkumar S, Martinez-Cajas JL, Claessens C, Lambert G, et al. Head-to-head comparison of accuracy of a rapid point-of-care HIV test with oral versus whole-blood specimens: a systematic review and meta-analysis. Lancet Infect Dis. 2012;12(5):373-80.

34. World Health Organization. HIV assays: laboratory performance and other operational characteristics. Rapid Diagnostic tests (Combined detection of HIV-1/2 antibodies and discriminatory detection of HIV-1 and HIV-2 antibodies). Report 18. 2015.

35. Johnson CC, Fonner V, Sands A, Ford N, Obermeyer CM, Tsui S, et al. To err is human, to correct is public health: a systematic review examining poor quality testing and misdiagnosis of HIV status. Journal of the International Aids Society. 2017;20.

36. Sibanda E, Neuman M, Tumushime M, Hatzold K, Watadzaushe C, Mutseta MN, et al. Linkage to care after HIV self-testing in Zimbabwe: a cluster-randomized trial. 25th Conference on Retroviruses and Opportunistic Infections; 2018 Mar 4-7; Boston, USA.

37. Choko AT, MacPherson P, Webb EL, Willey BA, Feasy H, Sambakunsi R, et al. Uptake, accuracy, safety, and linkage into care over two years of promoting annual self-testing for HIV in Blantyre, Malawi: a community-based prospective study. Plos Med. 2015;12(9):e1001873.

38. Choko AT, MacPherson P, Webb EL, Ball H, Sambakunsi R, Mdolo A, et al., editors. One Year Outcomes Following Community-Based HIV Self-Testing: A Prospective Study in Malawi. 21st Conference on Retroviruses and Opportunistic Infections; 2014 Mar 6; Boston, USA.

39. Kennedy CE, Fonner VA, Sweat MD, Okero FA, Baggaley R, O'Reilly KR. Provider-initiated HIV testing and counseling in low- and middle-income countries: a systematic review. AIDS Behav,. 2013;17(5):1571-90.

40. Cremin I, Nyamukapa C, Sherr L, Hallett TB, Chawira G, Cauchemez S, et al. Patterns of self-reported behaviour change associated with receiving voluntary counselling and testing in a longitudinal study from Manicaland, Zimbabwe. AIDS Behav,. 2010;14(3):708-15.

41. Oldenburg C, Chanda MM, Ortblad KF, Mwale M, Chongo S, Kamungoma N, et al. Effect of HIV self-testing on the number of sexual partners among female sex workers in Zambia: A randomized controlled trial. AIDS. 2018.

42. Johnson CC, Kennedy C, Fonner V, Siegfried N, Figueroa C, Dalal S, et al. Examining the effects of HIV self-testing compared to standard HIV testing services: a systematic review and meta-analysis. Journal of the International Aids Society. 2017;20.

43. Mwenge L, Sande L, Mangenah C, Ahmed N, Kanema S, d’Elbée M, et al. Costs of facility-based HIV testing in Zambia, Malawi and Zimbabwe. PLoS ONE 2017;10(12):e0185740.

44. Hatzold K. Cost-effectiveness question for Zimbabwe. In: Cambiano V, editor. 2016.

45. Clinton Health Access Initiative. ARV Market report; The state of the antiretroviral drug market in low- and middle-income countries, 2016-2021. 2017.

46. Eaton JW, Menzies NA, Stover J, Cambiano V, Chindelevitch L, Cori A, et al. Health benefits, costs, and cost-effectiveness of earlier eligibility for adult antiretroviral therapy and expanded treatment coverage: a combined analysis of 12 mathematical models. Lancet Glob Health. 2014;2(1):e23-34.

47. Hyle EP, Jani IV, Lehe J, Su AE, Wood R, Quevedo J, et al. The clinical and economic impact of point-of-care CD4 testing in mozambique and other resource-limited settings: a cost-effectiveness analysis. Plos Med. 2014;11(9):e1001725.

48. Keebler D, Revill P, Braithwaite S, Phillips A, Blaser N, Borquez A, et al. Cost-effectiveness of different strategies to monitor adults on antiretroviral treatment: a combined analysis of three mathematical models. Lancet Glob Health. 2014;2(1):e35-43.

49. Tagar E, Sundaram M, Condliffe K, Matatiyo B, Chimbwandira F, Chilima B, et al. Multi-country analysis of treatment costs for HIV/AIDS (MATCH): Facility-level ART unit cost analysis in Ethiopia, Malawi, Rwanda, South Africa and Zambia. Plos One. 2014;9(11).

50. Siapka M, Remme M, Obure CD, Maier CB, Dehne KL, Vassall A. Is there scope for cost savings and efficiency gains in HIV services? A systematic review of the evidence from low- and middle-income countries. B World Health Organ. 2014;92(7):499-511.

51. Njeuhmeli E, Kripke K, Hatzold K, Reed J, Edgil D, Jaramillo J, et al. Cost analysis of integrating the PrePex medical device into a voluntary medical male circumcision program in Zimbabwe. PLoS One. 2014;9(5):e82533.

52. Salomon JA, Vos T, Hogan DR, Gagnon M, Naghavi M, Mokdad A, et al. Common values in assessing health outcomes from disease and injury: disability weights measurement study for the Global Burden of Disease Study 2010. Lancet. 2012;380(9859):2129-43.
